# Recombinant rabies virus with the glycoprotein fused with a DC-binding peptide is an efficacious rabies vaccine

**DOI:** 10.18632/oncotarget.23160

**Published:** 2017-12-11

**Authors:** Yachun Zhang, Ming Zhou, Yingying Li, Zhaochen Luo, Huanchun Chen, Min Cui, Zhen F. Fu, Ling Zhao

**Affiliations:** ^1^ State Key Laboratory of Agricultural Microbiology, Huazhong Agricultural University, Wuhan 430070, China; ^2^ College of Veterinary Medicine, Huazhong Agricultural University, Wuhan 430070, China; ^3^ Department of Pathology, University of Georgia, Athens, GA 30602, USA

**Keywords:** rabies virus, dendritic cells, DC-binding peptides, rabies vaccine

## Abstract

Our previous studies demonstrated that recruiting and/or activating dendritic cells (DCs) enhanced the immunogenicity of recombinant rabies viruses (rRABV). In this study, rRABV LBNSE with a small DC-binding peptide (designated as rLBNSE-DCBp) or a negative control peptide (designated as rLBNSE-DCCp) fused to the glycoprotein (G) was constructed and rescued. As expected, significantly more activated DCs were detected in rLBNSE-DCBp-immunized mice than those immunized with rLBNSE or rLBNSE-DCCp. Subsequently, significantly more generation of T_FH_ and GC B cells were observed in rLBNSE-DCBp immunized mice than those in rLBNSE or rLBNSE-DCCp-immunized mice. In addition, significantly higher levels of virus neutralizing antibodies (VNAs) were observed in mice immunized with rLBNSE-DCBp than those immunized with rLBNSE or rLBNSE-DCCp, resulting in a better protection of rLBNSE-DCBp immunized mice against the lethal challenge. Taken together, our results suggest that rRABV with G fused with DCBp is a promising rabies vaccine candidate.

## INTRODUCTION

Rabies is a zoonotic viral disease that causes more than 59,000 human deaths annually all over the world [1]. Its causative pathogen, rabies virus (RABV), is a neurotropic virus, consisting of five genes nucleoprotein (N), phosphoprotein (P), matrix protein (M), glycoprotein (G) and the viral RNA polymerase (L) [2]. From the site of entry, RABV moves fast along the peripheral nervous system and reach the central nervous system (CNS) eventually. It is almost a death sentence once clinical signs appear [3]. Although rabies is fatal, it can be prevented by appropriate vaccination in humans and animals [4]. After immunization, the immune system will be activated and antibodies produced to neutralize the virus. Since the first introduction in 1885, vaccination has become the most effective way to protect people from rabies [5]. Millions of people are vaccinated globally and it is estimated that this saves more than 250,000 people from dying of rabies every year [6].

Since vaccination is critical for rabies control, various efforts have been made to improve the immunogenicity of current rabies vaccines, such as expressing multiple G proteins and immune-stimulating molecules [7]. Our previous studies have shown a strong correlation between dendritic cell (DC) activation and RABV neutralizing antibody generation [8, 9]. It is known that mature DCs are the most efficient antigen-presenting cells (APCs) [10, 11]. They are capable of transforming antigens into immunogens and inducing expression of molecules to initiate the downstream adaptive immune response [12, 13]. Thus, the interaction of DCs with vaccines most often results in the yield of neutralizing antibodies which are protective against pathogenic RABV [14, 15], indicating that DCs play an important role in RABV immunogenicity.

In previous studies, several cytokines or chemokines have been demonstrated to be capable of enhancing DC activation when over-expressed by recombinant RABV (rRABV) [8, 9, 16–18]. However, over-expression of these cytokines or chemokines may provide other functions, even some side effects, beyond DC activation. It thus needs further investigate if solely increasing the binding of rRABV to DCs is sufficient to enhance RABV immunogenicity. Coincidentally, it was reported that a 12-mer DC-binding peptide (named DCBp thereafter) derived from a phage display library could improve the taken up efficiency of hepatitis C virus NS3 by DCs to enhance the immunogenicity. The DCBp could bound distinct and saturable DC surface epitopes ( the ligand is currently under intensive scrutiny) with the dissociation constants in the nanomolar range [19]. Thus, a DC-binding strategy was employed in this study to enhance immunogenicity of RABV. A recombinant RABV expressing a DCBp was constructed. It was found that rLBNSE-DCBp could facilitate the recruitment and activation of DCs, leading to a robust virus neutralizing antibodies (VNA) production and enhanced protection against the lethal challenge of rabies. Our study provides a promising strategy to enhance the efficiency of rabies vaccines.

## RESULTS

### Construction and characterization of the recombinant RABV expressing a DC-binding peptide (DCBp)

**Figure 1:**
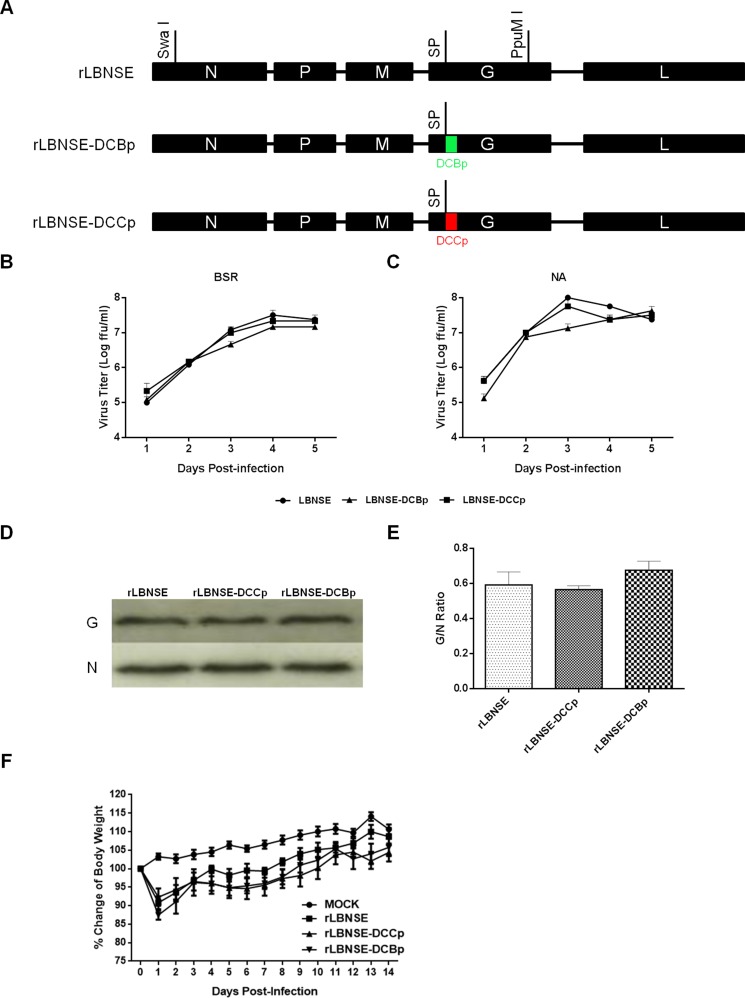
Construction and *in vitro* characterization of different rRABVs (**A**) Schematic diagram for the construction of rRABVs. A DC-binding peptide (DCBp) and a control peptide (DCCp) were fused with G protein next to the signal peptide. The growth kinetics of rRABVs in BSR (**B**) and NA cells (**C**) were determined. Briefly, BSR or NA cells were infected with different rRABVs at multiplicity of infection (MOI) of 0.01. At days 1, 2, 3, 4 and 5, the supernatants were collected and virus titers were determined to depict the growth kinetics; (**D**) Detection of the expression of G and N proteins in different rRABVs infected cells by western blot. BSR cells were infected with different rRABVs at MOI of 0.01, and the Western blot was carried out to detect the expression of G and N proteins in infected cells. (**E**) The G/N ratio in different rRABVs infected cells. The ratio was calculated according to the intensity detected by Western blot. (**F**) Pathogenicity of different rRABVs in mice. Groups of 10 ICR mice (6–8-week-old, female) were infected i.c. with 1.6 × 10^6^ FFU of rLBNSE, rLBNSE-DCCp or rLBNSE-DCBp or mock in the same volume of DMEM, and body weights were monitored daily for 2 weeks. Data was obtained from all 10 mice in each group and measured as mean values ± SEM.

To further determine if increasing the binding efficiency of the rRABV to DCs is sufficient to enhance the immunogenicity of RABV, a small DC-binding peptide (DCBp) and a control peptide (DCCp, do not bind to DCs) that characterized in previous studies were inserted after the signal peptide of G protein of LBNSE strain by fusion PCR, and designated as rLBNSE-DCBp and rLBNSE-DCCp, respectively (Figure 1A). The rRABVs were rescued as described previously [22] and verified by RT-PCR and sequencing. Multi-step growth curves on BSR (Figure 1B) and NA (Figure 1C) cells were depicted, and the results show that the growth curves of rLBNSE-DCBp and rLBNSE-DCCp were similar to the parent virus rLBNSE, indicating that the insertion of DCBp or DCCp into G did not affect the viral replication *in vitro*. Furthermore, due to the insertion site located next to the signal peptide of G protein, the expression level of G protein was measured by Western blot. As shown in Figure 1D, the G protein expression in rLBNSE-DCBp or rLBNSE-DCCp infected cells were similar to that in cells infected with rLBNSE, and no significant difference on G/N ratio was observed (Figure 1E), suggesting that insertion of DCBp or DCCp did not affect G protein expression.

In addition, the pathogenicity of the rRABVs was evaluated by measuring the mouse body weight changes after inoculation with 1.6 × 10^6^ FFU of each rRABV through intracranial (i.c.) route. No significant difference in body weight change was found among mice infected with rLBNSE, rLBNSE-DCBp, or rLBNSE-DCCp (Figure 1F), indicating that the insertion of DCBp or DCCp did not affect the viral pathogenicity in mice.

### Activation of bone marrow-derived DCs by rRABVs *in vitro*

**Figure 2:**
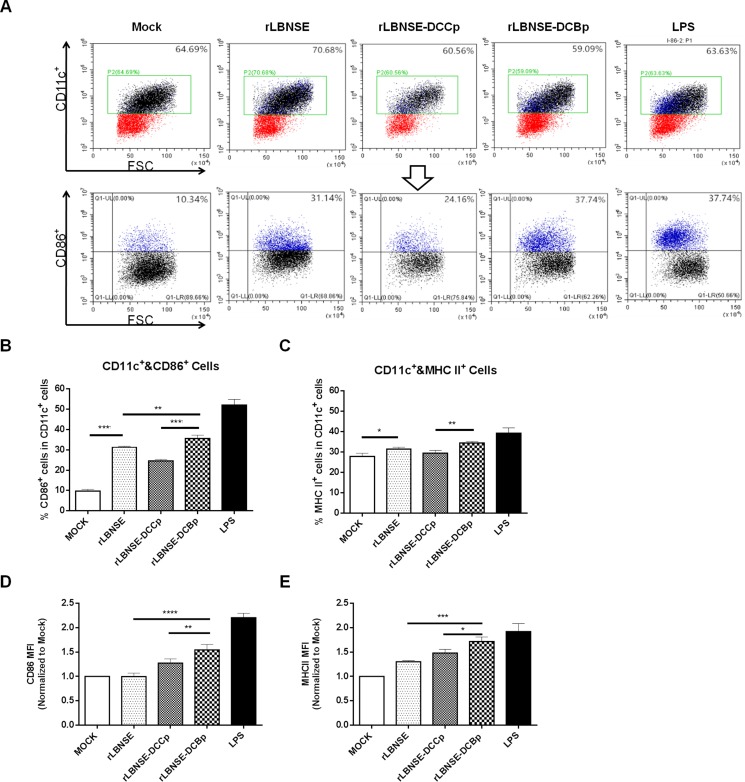
Activation of bone marrow-derived DCs after infection with different rRABVs Bone marrow cells were harvested from BALB/c mice, and DC precursors were cultured with GM-CSF. LPS was used as positive control, and the medium from untreated cells (mock) was used as negative controls. (**A**) Representative gating strategy for DCs. (**B**) Percentages of CD11c^+^ and CD86^+^ activated DCs after infection with different rRABVs. (**C**) CD11c^+^ and MHC II^+^ activated DCs after infection with different rRABVs. (**D**) and (**E**) The mean fluorescence intensities (MFI) of CD86^+^ and MHC II^+^ staining on CD11c^+^ cells, respectively. The results were normalized to the results of the mock-infected cells. Data were the means from three independent experiments (^*^P < 0.05; ^**^P < 0.01; ^***^P < 0.001).

To investigate if insertion of DCBp targets, binds and finally activates DCs *in vitro*, DCs were prepared from mouse bone marrow, and the purity checked by FACS were about 90% (data not shown). Then the prepared DCs were incubated with each rRABV, and lipopolysaccharide (LPS) was used as a positive control. A representative gating strategy of DCs (CD11c^+^ and CD86^+^) was as shown in Figure 2A. As expected, significantly higher percentage of CD86^+^ (Figure 2B) or MHC II^+^ (Figure 2C) cells in CD11c^+^ cells were detected in cells incubated with rLBNSE-DCBp or rLBNSE than those incubated with rLBNSE-DCCp or DMEM (mock). In addition, significantly more activated DCs (CD11c^+^ and CD86^+^) were observed in rLBNSE-DCBp incubated cells than those incubated with rLBNSE (Figure 2B). Furthermore, to evaluate differences in the levels of CD86 and MHC II expression within the total activated DC population, mean fluorescence intensities (MFI) were calculated among cells incubated with different rRABVs by normalizing to those in mock infected cells. As shown in Figure 2D and 2E, both the MFI of CD86 and MHC IIwere significantly higher in cells incubated with rLBNSE-DCBp than those incubated with rLBNSE or rLBNSE-DCCp. Overall, rLBNSE-DCBp could activate significantly more DCs than rLBNSE-DCCp or rLBNSE *in vitro*.

### Activation of DCs after immunization with different rRABVs in mice

**Figure 3:**
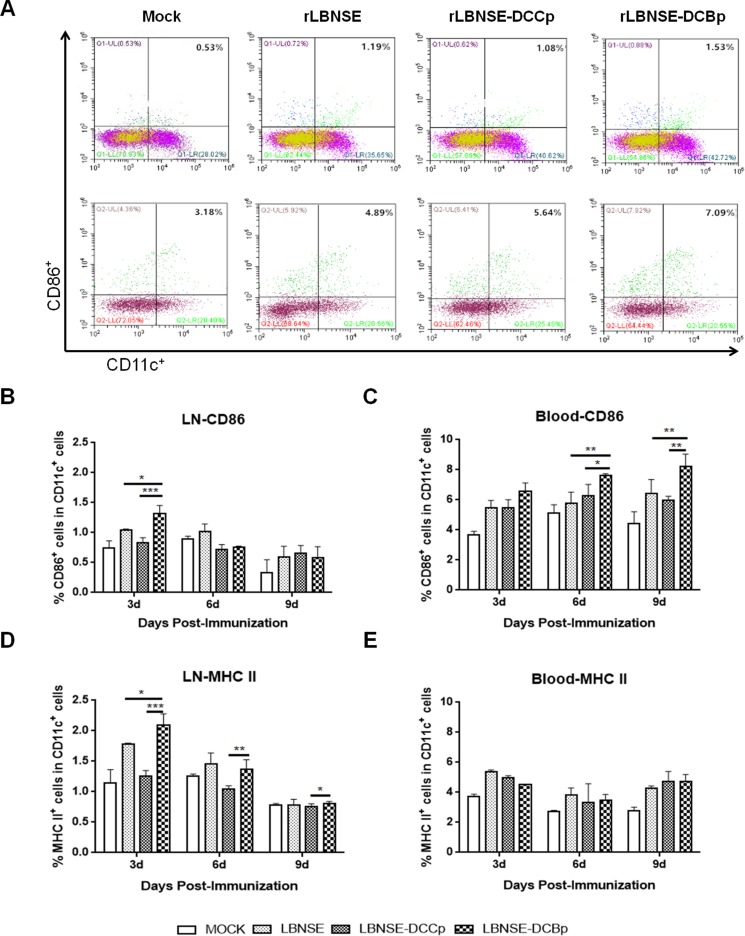
DC activation in mice immunized with different rRABVs BABLB/c mice were immunized with 1 × 10^6^ FFU of rRABVs or DMEM. The lymph nodes (LN) and blood samples were collected at 3, 6 and 9 dpi. Single cell suspensions prepared from the lymph nodes and blood were analyzed for the presence of DCs (CD11c^+^ and CD86^+^, or CD11c^+^ and MHC II^+^). (**A**) Representative gating strategy for DCs in blood or inguinal lymph samples. (**B**) and (**C**) Percentages of CD11c^+^ and CD86^+^ activated DCs in LN and blood samples of immunized mice respectively. (**D**) and (**E**) Percentages of CD11c^+^ and MHCII^+^ activated DCs in LN and blood samples of immunized mice respectively. Data are the means from three independent experiments (^*^P < 0.05; ^**^P < 0.01; ^***^P < 0.001).

To examine whether rLBNSE-DCBp could activate more DCs *in vivo*, groups of BALB/c mice ( *n =* 5) were immunized with 10^6^ FFU rRABV or mock immunized with DMEM by intramuscular (i.m.) route. Blood and inguinal lymph nodes were collected at 3, 6 and 9 days post-immunization (dpi), and single cell suspension was prepared for the detection of activated DCs (CD11c^+^ and CD86^+^ or MHC II^+^) via flow cytometry. The representative gating strategy for activated DCs (CD11c^+^ and CD86^+^) from blood or lymph nodes was shown in Figure 3A. Significantly more CD11c^+^ and CD86^+^ DCs were detected in lymph nodes of mice immunized with rLBNSE-DCBp than those immunized with rLBNSE-DCCp or rLBNSE at 3 dpi (Figure 3B), while the significant more CD11c^+^ and CD86^+^ DCs were observed at 6 and 9 dpi in the blood (Figure 3C). In addition, significantly more CD11c^+^ and MHC II^+^ DCs were found in lymph nodes of mice immunized with rLBNSE-DCBp than those immunized with rLBNSE-DCCp or rLBNSE at 3 dpi (Figure 3D), while no significant difference was detected in the blood (Figure 3E). The above data illustrates that rLBNSE-DCBp could recruit more DCs both in the blood and inguinal lymph nodes in immunized mice than the parent virus.

### Formation of T_FH_ and germinal center (GC) B cells in mice immunized with different rRABVs

**Figure 4:**
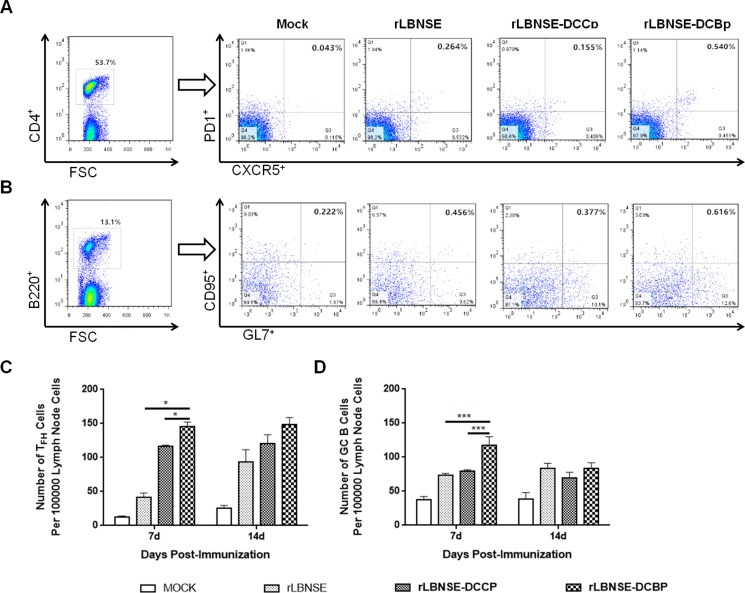
Generation of T_FH_ and GC B cells in different rRABVs immunized mice BALB/c mice were infected with 1 × 10^6^ FFU of different rRABVs or mock immunized with DMEM by i.m. route. The inguinal lymph nodes were collected at 7 and 14 dpi. Single cell suspensions prepared from the inguinal lymph nodes were analyzed for the generation of GC B (B220^+^GL7^hi^CD95/Fas^hi^) and T_FH_ cells (CD4^+^CXCR5^hi^PD1^lo^). (**A**) and (**B**) Representative gating strategies for T_FH_ and GC B cells respectively. (**C**) and (**D**) Percentages of T_FH_ and GC B cells in inguinal LNs from mice immunized with different rRABVs respectively. Data are the means from three independent experiments (^*^P < 0.05; ^**^P < 0.01; ^***^P < 0.001).

After the capture of antigen by DCs, the antigen is then processed and presented to T cells, and CD4^+^ naïve T cells differentiate into several subtypes, such as follicular helper T (T_FH_) cells, which play an important role in the formation of the GC and generation of GC B cells with high affinity for the antigen. Therefore, the generation of T_FH_ and GC B cells were detected in the lymph nodes of mice immunized with 10^6^ FFU of rRABVs at 7 and 14 dpi. Single cell suspension was prepared, and GC B cells (B220^+^GL7^hi^CD95/Fas^hi^) and T_FH_ cells (CD4^+^CXCR5^hi^PD1^hi^) analyzed through Flow cytometry. A representative gating strategy for T_FH_ (Figure 4A) and GC B (Figure 4B) cells were shown. As expected, significantly more T_FH_ and GC B cells were detected in lymph nodes of mice immunized with rLBNSE-DCBp than those immunized with rLBNSE-DCCp or rLBNSE at 7 dpi as shown in Figure 4C and 4D, respectively. Thus, the expression of DCBp could enhance the quantity of T_FH_ and GC B cells after immunization in mice.

### VNA induction and protection after immunization with different rRABVs in mice

**Figure 5:**
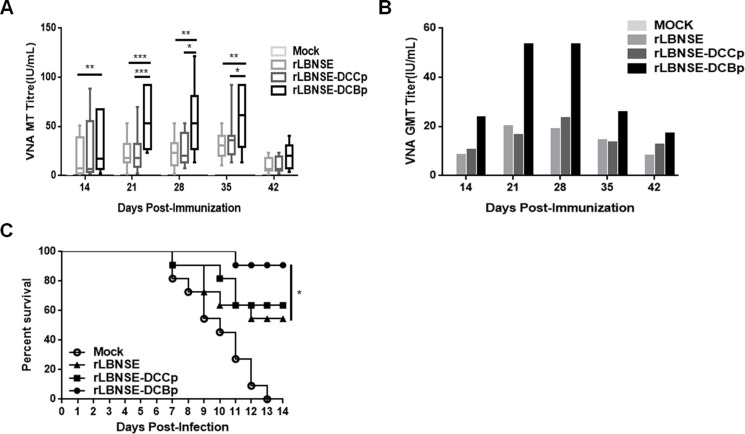
VNA production and survivorship after immunization with different rRABVs in mice Groups of ICR mice (*n* = 10) were immunized with 1 × 10^6^ FFU of rLBNSE, rLBNSE-DCCp, rLBNSE-DCBp or mock immunized with the same volume of DMEM by the i.m. route. At indicated time points, blood samples were collected and sera were separated for VNA test by FAVN. (**A**) and (**B**) The VNA titers and VNA geometric mean titers (GMT) determined at different indicated time points in mice immunized with different rRABVs. (**C**) Survivorship of mice immunized with different rRABVs. At 21 dpi, mice were challenged i.c. with 50 × LD_50_ of CVS-24 and observed twice a day for 21 days, and the numbers of survivors were recorded. Data are the means from three independent experiments (^*^P < 0.05; ^**^P < 0.01; ^***^P < 0.001).

To investigate if the enhanced generation of T_FH_ and GC B cells could increase the production of VNA, mice were immunized with 10^6^ FFU of rRABV or mock immunized with DMEM by the i.m. route, and blood samples were collected at different time points after vaccination for the measurement of VNA. As shown in Figure 5A, significantly higher levels of VNA titers were detected in rLBNSE-DCBp immunized mice than those immunized with rLBNSE at 14, 21, 28 and 35 dpi, or with rLBNSE-DCCp at 21, 28 and 35 dpi, respectively. The dynamics of geometric mean titers (GMT) of VNA were as presented in Figure 5B, and the highest GMT of VNA in rLBNSE-DCBp, rLBNSE-DCCp, and rLBNSE immunized mice were 53.30, 23.28, and 19.98 IU/mL reached at 28, 28, and 21 dpi, respectively. To further investigate if mice immunized with different rRABVs are protected from pathogenic RABV challenge, the immunized mice were challenged i.c. with 50 × LD_50_ of CVS-24 at 21 dpi and observed daily for 3 weeks. Consistent with the VNA titers, 90.91% of mice immunized with rLBNSE-DCBp were protected, which was significantly higher than those immunized with rLBNSE (54.55%), and higher than that for rLBNSE-DCCp immunized mice (63.64%) as shown in Figure 5C.

**Figure 6:**
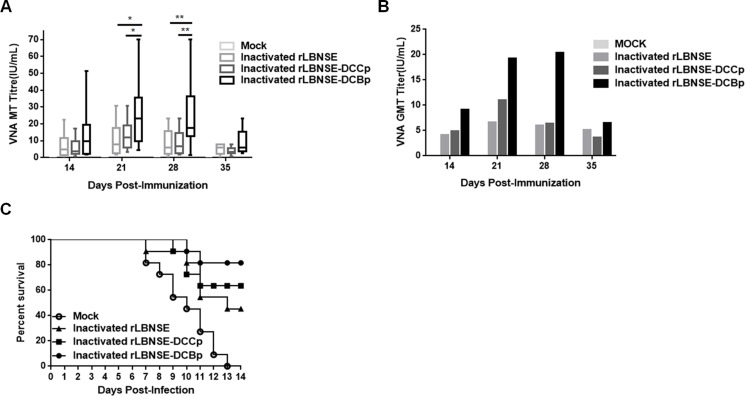
The efficiency used as inactivated vaccines in mice Groups of ICR mice (*n* = 10) were immunized i.m. with 1 × 10^7^ FFU of inactivated rLBNSE, rLBNSE-DCCp, rLBNSE-DCBp or the same volume of DMEM (mock). At indicated time points, blood samples were collected and sera were separated for VNA test by FAVN. (**A**) and (**B**) The VNA titers and VNA geometric mean titers (GMT) were determined at different indicated time points. (**C**) Survival ratio of mice immunized with different rRABVs. At 21 dpi, mice were challenged i.c. with 50 × LD_50_ of CVS-24 and observed twice a day for 21 days, and the survivorship was calculated. Data are the means from three independent experiments (^*^P < 0.05; ^**^P < 0.01; ^***^P < 0.001).

Since immunization with live-attenuated RABV could still present safety issues, inactivated rabies vaccine is currently widely used for vaccinating humans and domestic animals. Hence, to evaluate if expressing DCBp is efficient in stimulating immune responses and providing protection when used as a killed vaccine, 10^7^ FFU of rRABVs were inactivated by 4% paraformaldehyde and applied for mice vaccination by i.m. route. Blood samples were collected and sera were separated for VNA test. As shown in Figure 6A, significantly higher VNA titers were detected in mice immunized with inactivated rLBNSE-DCBp than those immunized with inactivated rLBNSE or rLBNSE-DCCp at 21 and 28 dpi. The highest GMT of VNA in inactivated rLBNSE-DCBp, rLBNSE-DCCp, and rLBNSE immunized mice were 20.36, 10.99, and 6.61 IU/mL reached at 28, 21, and 21 dpi, respectively, as shown in Figure 6B. At 21 dpi, mice were challenged with 50 × LD_50_ of CVS-24 by i.c. route, and consistent with the VNA titers, 81.82% of mice immunized with inactivated rLBNSE-DCBp could be protected, which was higher than those immunized with inactivated rLBNSE (45.45%) or rLBNSE-DCCp (63.64%) as shown in Figure 6C. Taken together, mice immunized i.m. with rLBNSE-DCBp (either live or inactivated) could induce higher level of VNA and provide a better protection than those immunized with parent virus rLBNSE or rLBNSE-DCCp, indicating that the rRABV expressing a DCBp (rLBNSE-DCBp) could be a promising rabies vaccine candidate, which provides a promising strategy for developing more efficient rabies vaccines.

## DISCUSSION

Rabies is a fatal disease and still poses a threat to public health; more than 15 million rabies-exposed patients require proper post exposure prophylaxis (PEP), which occur mainly in Southeast Asia and Africa [23]. According to WHO, 99% of human rabies cases are transmitted by dogs, therefore, the most cost-effective way to control human rabies is mass vaccination of dogs [24]. Hence, it is very meaningful to develop efficacious rabies vaccines used for dog vaccination. Our previous studies have demonstrated that expressing cytokines or chemokines could activate DCs to enhance the immunogenicity of RABV, suggesting that DCs play an important role in inducing protective immunity after vaccination. Recently, a DC-binding peptide (DCBp) that could enhance the uptake of antigen by DCs through elevating the binding efficiency between DCs and the antigen was introduced [19]. Following this rationale, an rRABV with the DCBp inserted in RABV G protein was constructed and rescued in this study. For the insertion site in G protein, a previous study demonstrated that the location where next to the signal peptide of G protein is a potential site for inserting exogenous peptides without affecting viral replication [25]. Consistent with this, rRABV with G protein fused with DCBp after the signal peptide was successfully rescued and did not affect the G protein expression or viral replication *in vitro*. In addition, DCBp inserted at this site most likely could be entirely or partially exposed on the viron surface since the insertion of DCBp could bind and activate significantly more DC as expected, and the exogenous peptide (GnRH) could induce the production of specific antibody [25].

DCs could help to induce potent Ag specific immunity in various conditions and it has been applied in clinic therapy [26, 27]. Our previous studies have found that expression of cytokines, which can activate DCs, improved the immunogenicity of rRABV [9, 28]. In the present study, as expected, more DCs were activated by rLBNSE-DCBp both *in vitro* and *in vivo*. Activated DCs could present antigens to CD4^+^ T cells through MHC II, which subsequently stimulate B cells to generate antigen-specific antibodies [29]. Indeed, higher VNA titers and better protection were observed in mice immunized with rLBNSE-DCBp, which is essential for a candidate vaccine. The mouse model was employed in the present study, it is not sure if it would work as well in the dog model. Interestingly, it has been demonstrated that this peptide (DCBp) could also recognize the conserved region of its ligand on canine DCs [30], suggesting that rLBNSE-DCBp could be used as a dog rabies vaccine, which warrants further investigation.

Vaccination of dogs is the most efficient way to prevent transmission of rabies to humans. Current RABV vaccines used in domestic animals are inactivated to avoid the possibility of reversion [31]. To reduce the possible safety risk, the rRABVs constructed in this study were also inactivated and investigated for the potential as killed vaccines. Higher VNA levels and survivorship were observed in rLBNSE-DCBp immunized mice, indicating the expression of DCBp by rRABV has the potential for using as a killed vaccine. It is known that virus treated with the paraformaldehyde usually became denature, and the protein structures would be destroyed, which means the spatial conformation of protein and peptide would totally changed. Based on this, we tried to analyze whether the inactivated DCBp could still imporve the DCs binding efficiency by using FACS, however, no significant difference on activation was observed between the BMDCs inoculated with inactivated rLBNSE-DCBp and rLBNSE-DCCp (data not shown), indicating that the higher VNA titer produced in mice immunized with inactivated rLBNSE-DCBp may not due to the improvement of DCs binding efficiency. The mechanism for this interesting phenomenon will be investigated in our future study.

In summary, rLBNSE-DCBp could induce a robust VNA response by enhancing DC binding and activation, and subsequently T_FH_ and GC B cell generation after vaccination, leading to a better protection against lethal virus challenge, which indicates that rLBNSE-DCBp has the potential to be exploited as a safe and efficacious rabies vaccine.

## MATERIALS AND METHODS

### Cells, viruses, antibodies, and animals

BSR cells, a cloned cell line derived from BHK-21 cells, were cultured in Dulbecco’s modified Eagle’s medium (DMEM) (Mediatech, Herndon, VA) supplemented with 10% fetal bovine serum (FBS) (Gibco, Grand Island, NY). rLBNSE is a rRABV constructed from SAD-B19 strain [20, 21] with two mutations at amino acid positions 194 and 333 of the G protein [17], which has been demonstrated to be attenuated in adult mice [32]. In this study, a recombinant rabies virus (rRABV) expressing a DC-binding peptide (FYPSYHSTPQRP), designated as rLBNSE-DCBp, was constructed, recovered and characterized as described previously [22]. Meanwhile, another rRABV expressing a small peptide (DCCp, EPIHPETTFTNN) that could not specifically bind to DCs was constructed, designated as rLBNSE-DCCp, and used as a negative control. Challenge virus CVS-24 was propagated in suckling mice brains. Fluorescein isothiocyanate (FITC)-conjugated antibody against the RABV N protein was purchased from Fujirebio Diagnostics, Inc. (Malvern, PA). Female BALB/c mice and ICR mice at the age of 6–8 weeks were purchased from the Center for Disease Control and Prevention of Hubei Province, China.

### Construction of rLBNSE-DCBp and rLBNSE-DCCp cDNA Clone

The rLBNSE-DCBp and rLBNSE-DCCp cDNA clone was generated from rLBNSE as described previously [22, 33]. Briefly, DCBp or DCCp was inserted next to the signal peptide of G protein by overlapping PCR. Then the amplified fragment was cloned into the vector pLBNSE digested with SwaI and PpuMI. Primers used in this study were listed in Table 1.

**Table 1:** Primers used in this study

Primer	Sequence (5′-3′)
1-F	5′-CCGATTTAAATAAAGCATACAAGTCAGTTTTGTCAGGCATGAG-3′
2-R	5′-GTCAGGTCCTAATATTATACCATTGAAAAACACCCCGTTCACATG-3′
Bp1-R	5′-*CTGGGGGGTGCTGTGGTAGCTGGGGTAGAA*CCCAAAACACAATGGAAAAAC-3′
Bp2-F	5′-*CCCAGCTACCACAGCACCCCCCAGAGGCCC*AAATTCCCTATTTACACGATAC-3′
Cp1-R	5′-*GGTGAAGGTGGTCTCGGGGTGGATGGGCTC*CCCAAAACACAATGGAAAAAC-3′;
Cp2-F	5′-*ATCCACCCCGAGACCACCTTCACC*AACAACAAATTCCCTATTTACACGATAC-3′

Note: *SwaI* and *PpuMI* sites are underlined and the nucleotides encoding DCBp or DCCp are italics.

### Rescue of rRABV and propagation of rRABVs

rLBNSE-DCBp and rLBNSE-DCCp were rescued as described previously [17]. Briefly, 2.0 μg of the full-length infectious clone, 0.5 μg of N-, 0.25 μg of P-, 0.1 μg of L- and 0.15 μg of G- helper plasmids were transfected into BSR cells using the SuperFect transfection reagent (Qiagen, Valencia, CA) according to the manufacturer’s protocol. The culture medium was discarded at 4 days post transfection and fresh medium replenished for further incubation (3 more days) at 34°C with 5% CO_2_. At 7 days post transfection, culture medium was harvested and tested for rescued virus using the FITC-conjugated antibody against RABV N protein.

### Virus titration

Virus titration was performed with the direct fluorescent antibody assay (dFA) in BSR cells as described previously [34]. The titrated virus was serial 10-fold diluted and incubated with BSR cells at 34°C for 48 h. Then the cells were fixed with 80% ice-cold acetone for 15 min and stained with FITC-conjugated anti-RABV N antibody at 37°C for 45 min. Antigen-positive foci were counted under a fluorescence microscope, and virus titer was calculated as fluorescent focus units per milliliter (FFU/mL). All titrations were carried out in quadruplicate.

### Growth kinetics of rRABVs *in vitro*

BSR or NA cells were cultured in six-well plates overnight and infected with each rRABV at an MOI of 0.01 for multi-step growth curves. After 1 h of incubation, the cells were washed three times with DMEM, and fresh DMEM supplemented with 2% FBS was added. The supernatant was harvested at 1, 2, 3, 4 and 5 days after infection. Virus titration was carried out and the growth kinetics were depicted according to the viral titers at each time point.

### Western blot

BSR cells were infected with rRABVs at an MOI of 0.01 for 72 h and then lysed with RIPA buffer (Thermo-Fisher Scientific). Proteins were resolved by 12% SDS-PAGE, and transferred onto a nitrocellulose (NC) membrane. Antibodies used in the Western blotting were mouse anti-G antibody (at a dilution of 1:5000), mouse anti-N antibody (at a dilution of 1:5000), mouse anti-β-actin antibody (SIGMA-ALDRICH, at a dilution of 1:5000) and goat anti-mouse secondary antibody labeled with horseradish peroxidase (HRP) (at dilution of 1:5000). Super Signal West Dura Extended Duration Substrate (Thermo-Fisher Scientific) was used for color development.

### VNA test

Virus-neutralizing antibody (VNA) titers were measured using the fluorescent-antibody virus neutralization (FAVN) test. Blood samples were collected and the sera were separated for VNA test. Serial 3-fold dilutions of serum (50 μL) or the same volume of reference serum (obtained from the National Institute for Biological Standards and Control, Herts, United Kingdom) in 100 μL of DMEM, and 50 μL of rabies challenge virus (CVS-11) suspension (50 to 200 FFU) were added in 96-well plates, and then incubated at 37°C for 1 h. BSR cells (2 × 10^4^) suspension (50 μL) were added to each well, and the plates were incubated at 34°C for 60 h. The dFA was then carried out in BSR cells, and the VNA titers were expressed as FFU/mL by normalized to the titer of the reference serum. All titrations were carried out in quadruplicate.

### Isolation of bone marrow-derived DCs

Bone marrow-derived DCs were isolated as described previously [35, 36]. Briefly, 6–8 weeks old BALB/c mice were euthanized and the femur was separated. Then bone marrow was collected and cultured in RPMI 1640 supplemented with 10% FBS and 20 ng/mL recombinant mouse GM-CSF at the density of 2 × 10^5^/mL. At 1, 3, and 5 days post cultivation, half of the medium was removed and fresh DC medium was replenished. The cells were collected and cultured in 12 well plates (10^6^/mL) at 7 days post cultivation, and the DCs were ready for the study 2 days later.

### Flow cytometry

Draining lymph nodes and blood were collected at each time point after immunization. Single-cell suspension was prepared at 10^6^ cells/mL in 0.2% BSA and stained with antibodies against CD11c, CD86, MHC II, B220, GL7, CD95, CD4, CXCR5 and PD1 at 4°C for 30 min. Cells were subsequently washed twice with PBS and fixed in 4% paraformaldehyde for 30 min. Flow cytometry was performed on LSR-II flow cytometer (BD Bioscience) and analyzed by FlowJo software. Data represents samples completed in duplicate (*n =* 3 mice) [37].

### Mice immunization and challenge experiment

Groups of 11 female six to eight-week-old ICR mice were immunized i.m. with 10^6^ FFU of each rRABV or DMEM in a volume of 100 μL by i.m. route. At 21 days post immunization (dpi), mice were intracerebrally (i.c.) challenged with 50 mouse intracerebral lethal dose 50 (MICLD_50_) of CVS-24 in a volume of 40 μL and observed daily for 3 weeks.

### Ethics statement

The animal experiments were carried out in strict accordance with the protocols approved by The Scientific Ethics Committee of Huazhong Agricultural University (permit number: HZAUMO-2015-022). The animal care and maintenance were in compliance with the recommendations in the Regulations for the Administration of Affairs Concerning Experimental Animals made by the Ministry of Science and Technology of China.

### Statistical analysis

Kaplan-Meier survival curves were analyzed by the log rank test; statistical analyses of the other data were determined by one-way ANOVA with GraphPad prism software. For all tests, the following notations were used to indicate significant differences between groups: ^*^P < 0.05; ^**^P < 0.01; ^***^P < 0.001.

## References

[1] Organization WH

[2] Finke S, Conzelmann KK (2005). Replication strategies of rabies virus. Virus research.

[3] Jackson AC

[4] Abela-Ridder B (2015). Rabies: 100 per cent fatal, 100 per cent preventable. Veterinary Record.

[5] Nunnally BK, Turula VE, Sitrin RD

[6] Wunner WH, Briggs DJ (2010). Rabies in the 21 st century. PLoS Negl Trop Dis.

[7] Liu X, Yang Y, Sun Z, Chen J, Ai J, Dun C, Fu ZF, Niu X, Guo X (2014). A recombinant rabies virus encoding two copies of the glycoprotein gene confers protection in dogs against a virulent challenge. PloS one.

[8] Zhao L, Toriumi H, Wang H, Kuang Y, Guo X, Morimoto K, Fu ZF (2010). Expression of MIP-1α (CCL3) by a recombinant rabies virus enhances its immunogenicity by inducing innate immunity and recruiting dendritic cells and B cells. Journal of virology.

[9] Zhou M, Zhang G, Ren G, Gnanadurai CW, Li Z, Chai Q, Yang Y, Leyson CM, Wu W, Cui M, Fu ZF (2013). Recombinant rabies viruses expressing GM-CSF or flagellin are effective vaccines for both intramuscular and oral immunizations.

[10] Moser M, Murphy KM (2000). Dendritic cell regulation of TH1-TH2 development. Nature immunology.

[11] Quaratino S, Duddy LP, Londei M (2000). Fully competent dendritic cells as inducers of T cell anergy in autoimmunity. Proceedings of the National Academy of Sciences.

[12] Kapsenberg M, Hilkens C, Wierenga E, Kalinski P (1999). The paradigm of type 1 and type 2 antigen-presenting cells. Implications for atopic allergy. Clinical & Experimental Allergy.

[13] Vieira PL, de Jong EC, Wierenga EA, Kapsenberg ML, Kaliński P (2000). Development of Th1-inducing capacity in myeloid dendritic cells requires environmental instruction. The Journal of Immunology.

[14] (1978). Immunoglobulin (IgG) and (IgM) antibody responses to rabies vaccine. Journal of General Virology.

[15] Johnson N, Cunningham AF, Fooks AR (2010). The immune response to rabies virus infection and vaccination. Vaccine.

[16] Zhou M, Wang L, Zhou S, Wang Z, Ruan J, Tang L, Jia Z, Cui M, Zhao L, Fu ZF (2015). Recombinant rabies virus expressing dog GM-CSF is an efficacious oral rabies vaccine for dogs. Oncotarget.

[17] Wen Y, Wang H, Wu H, Yang F, Tripp RA, Hogan RJ, Fu ZF (2011). Rabies virus expressing dendritic cell-activating molecules enhances the innate and adaptive immune response to vaccination. Journal of virology.

[18] Wang Z, Li M, Zhou M, Zhang Y, Yang J, Cao Y, Wang K, Cui M, Chen H, Fu ZF (2017). A Novel Rabies Vaccine Expressing CXCL13 Enhances Humoral Immunity by Recruiting both T Follicular Helper and Germinal Center B Cells. Journal of virology.

[19] Curiel TJ, Morris C, Brumlik M, Landry SJ, Finstad K, Nelson A, Joshi V, Hawkins C, Alarez X, Lackner A (2004). Peptides identified through phage display direct immunogenic antigen to dendritic cells. The Journal of Immunology.

[20] Conzelmann KK, Cox JH, Schneider LG, Thiel HJ (1990). Molecular cloning and complete nucleotide sequence of the attenuated rabies virus SAD B19. Virology.

[21] Rasalingam P, Rossiter JP, Mebatsion T, Jackson AC (2005). Comparative pathogenesis of the SAD-L16 strain of rabies virus and a mutant modifying the dynein light chain binding site of the rabies virus phosphoprotein in young mice. Virus research.

[22] Wu X, Smith TG, Franka R, Wang M, Carson WC, Rupprecht CE (2014). The feasibility of rabies virus-vectored immunocontraception in a mouse model. Trials in Vaccinology.

[23] Bourhy H, Dautry-Varsat A, Hotez PJ, Salomon J (2010). Rabies, still neglected after 125 years of vaccination. PLoS Negl Trop Dis.

[24] Lembo T, Partners for Rabies Prevention (2012). Blueprint for rabies prevention and control. PLoS Negl Trop Dis.

[25] Wu X, Franka R, Svoboda P, Pohl J, Rupprecht CE (2009). Development of combined vaccines for rabies and immunocontraception. Vaccine.

[26] Hsu FJ, Benike C, Fagnoni F, Liles TM, Czerwinski D, Taidi B, Engleman EG, Levy R (1996). Vaccination of patients with B–cell lymphoma using autologous antigen–pulsed dendritic cells. Nature medicine.

[27] Amodio G, Annoni A, Gregori S (2015). Dendritic Cell Immune Therapy to Break or Induce Tolerance. Current Stem Cell Reports.

[28] Wu X, Smith TG, Rupprecht CE (2011). From brain passage to cell adaptation: the road of human rabies vaccine development. Expert Rev Vaccines.

[29] Cho KA, Kim JY, Kim HS, Ryu KH, Woo SY (2012). Tonsil-derived mesenchymal progenitor cells acquire a follicular dendritic cell phenotype under cytokine stimulation. Cytokine.

[30] Owen JL, Sahay B, Mohamadzadeh M (2013). New generation of oral mucosal vaccines targeting dendritic cells. Current opinion in chemical biology.

[31] Yendo AC, de Costa F, Cibulski SP, Teixeira TF, Colling LC, Mastrogiovanni M, Soulé S, Roehe PM, Gosmann G, Ferreira FA (2016). A rabies vaccine adjuvanted with saponins from leaves of the soap tree (Quillaja brasiliensis) induces specific immune responses and protects against lethal challenge. Vaccine.

[32] Faber M, Faber ML, Li J, Preuss MA, Schnell MJ, Dietzschold B (2007). Dominance of a nonpathogenic glycoprotein gene over a pathogenic glycoprotein gene in rabies virus. Journal of virology.

[33] Schnell MJ, Mebatsion T, Conzelmann KK (1994). Infectious rabies viruses from cloned cDNA. The EMBO journal.

[34] Klimstra WB, Ryman KD, Johnston RE (1998). Adaptation of Sindbis virus to BHK cells selects for use of heparan sulfate as an attachment receptor. Journal of virology.

[35] Lutz MB, Kukutsch N, Ogilvie AL, Rößner S, Koch F, Romani N, Schuler G (1999). An advanced culture method for generating large quantities of highly pure dendritic cells from mouse bone marrow. Journal of immunological methods.

[36] Gilboa E (2007). DC-based cancer vaccines. Journal of Clinical Investigation.

[37] Dorfmeier CL, Lytle AG, Dunkel AL, Gatt A, McGettigan JP (2012). Protective vaccine-induced CD4+ T cell-independent B cell responses against rabies infection. Journal of virology.

